# Living libraries: Nurse integration in interprofessional homeless health care team

**DOI:** 10.1111/phn.12561

**Published:** 2018-11-22

**Authors:** Melanie A. Mariano, Monica J. Harmon

**Affiliations:** ^1^ School of Nursing University of Pennsylvania, Hospital of the University of Pennsylvania Philadelphia Pennsylvania; ^2^ School of Nursing Villanova University Philadelphia Pennsylvania

**Keywords:** community health, community health services, community‐based nursing, interprofessional, preventive health services

## Abstract

**Background:**

Despite an increase in national health care service utilization, entry into the health care system remains inequitable. This disparity in health care access disproportionately affects those experiencing homelessness. Because the homeless population faces significant financial and nonfinancial barriers, health care system engagement with these individuals must be reconsidered.

**Objective:**

This article will describe the piloting of an interprofessional model within an urban library to address barriers to health care access that homeless individuals face.

**Design:**

The library's unique status as a community hub presents an opportunity for partnership in addressing this population's health care access issues. This community‐based model is the first recorded to utilize three distinct professions—nursing, social work, and library science—in a public library.

**Results and Conclusions:**

The implementation of this pilot project resulted in a high retention rate of referrals to community health services for those unstably housed and facilitated a system of warm transfers. Although opportunities to improve generalizability exist, this initiative sets the stage for discussion around co‐location of health and social services in a nontraditional community‐based setting to achieve equitable access to health care.

## 1 INTRODUCTION AND BACKGROUND

Both health care expenditures and utilization have increased in the United States (Altarum Institute, 2017). Despite this growth, disparities to health care access exist based on race, ethnicity, socioeconomic status, gender identity, disability status, and location (Agency for Healthcare Research & Quality, 2015). Homeless populations face significant barriers to health care access (Fazel, Geddes, & Kushel, 2014).

Homelessness and barriers to health care entry are complex issues. On a single night in 2017, 553,742 people were experiencing homelessness nationwide, an increase for the first time in 7 years (U.S. Department of Housing & Urban Development, 2017). In a 2017 point‐in‐time count in Philadelphia, 5,693 persons were found to be experiencing homelessness (Office of Homeless Services [OHS], 2017), including 825 families with 1,508 children and 242 parenting youth, aged 18–24 (OHS, 2017). With these numbers, Philadelphia found itself on the top 10 list of U.S. cities with the highest rates of homelessness. There is strong evidence to support that health care of homeless individuals is more costly with their high utilization of the emergency department, more expensive hospital stays, added advanced chronic comorbidities, and greater likelihood of health care needs going unmet (Donley & Wright, 2017; Mitchell, Leon, Byrne, Lin, & Bharel, 2017).

To manage these health care needs, individuals require access to primary care services. The challenge is that individuals first have to navigate a host of issues that challenge easy access to services, especially for those experiencing homelessness (Fazel et al., 2014). The financial barrier is apparent in urban areas such as Philadelphia, as nearly 20% of all Philadelphians have forgone medically necessary care due to cost (Philadelphia Department of Public Health, 2014).

Cost‐related barriers are not the only barriers to health care access. Insurance status, mental health problems, substance abuse, and medical comorbidities all contribute to the inability to obtain necessary medical care (Corrigan, Pickett, Kraus, & Schmidt, 2015). Barriers that result in no‐show appointments further exacerbate the financial burden on the health care system (Jabrera Mesa, Morales Asencio, Rivas Ruiz, & Porras González, 2017).

To address these barriers, the City of Philadelphia provides case management support to those enrolled in housing programs or rehabilitation and treatment programs. Case managers work with clients to facilitate care coordination by scheduling appointments, arranging transportation, following up with clients, and providing support services to meet the comprehensive health needs of this population. However, the barriers to access remain for those who are waiting to gain access to these programs.

Limited available resources and the many issues surrounding health care access call for innovation to address these upstream factors and reach those without access to supportive services. Programs addressing limited health care access can be implemented with at‐risk individuals to mitigate factors associated with barriers to access. This paper will discuss a pilot project that utilizes a nontraditional community partner, an urban public library, to engage the homeless population and overcome barriers to access.

Public libraries can serve as low‐barrier locations to navigate around these health care access issues. In a nationwide survey, 73% of those aged 16 years and older say that libraries continue to serve as valued places to find health information (Horrigan, 2015). Over 42% of those who accessed the internet at the library reported doing so for health‐related searches (Horrigan, 2015). This number becomes increasingly important when looking at the volume of people who access the Free Library of Philadelphia (FLP). The FLP recognized that their high traffic and circulation aligned with the reported percentage of people using libraries for health information. With close to 5 million in‐person visits and 7 million online visits in 2016, the FLP was in a unique position to be the access point for health information and partner in health access navigation issues (Free Library of Philadelphia, 2016).

Like many urban libraries, the FLP is especially vital in its public service to vulnerable populations, including a large number of homeless patrons. The homeless population often used public libraries as a day shelter or place of refuge, a behavior observed in urban libraries across the nation (Johnson, 2015). The FLP, in particular, provided a neutral environment that was welcoming and receptive to the public while also providing warmth in the winter, cool temperatures in the summer, public restrooms, free computer and internet access, and open access to books and newspapers. In addition to the physical amenities, the FLP also serves as a social gathering place for those who are homeless, creating a sense of community, with patrons sharing resources and experiences. The landscape of the FLP facilitated its role as a community partner in addressing health care access issues.

To meet the health care access needs of the homeless population in a low‐barrier environment, the authors developed an initiative to bring a nurse into an interprofessional team at the library. A grant‐funded project, entitled Reaching HEALthy, supported the team presence in the FLP. The pilot initiative sought to promote health care expansion across libraries by addressing the confounding factors prohibiting adequate health care access. Reaching HEALthy was started to connect the volume of homeless people with needs, in a place where they already were, with the resources and access they needed for support.

### 1.1 Approach and Methods

**Figure 1:**
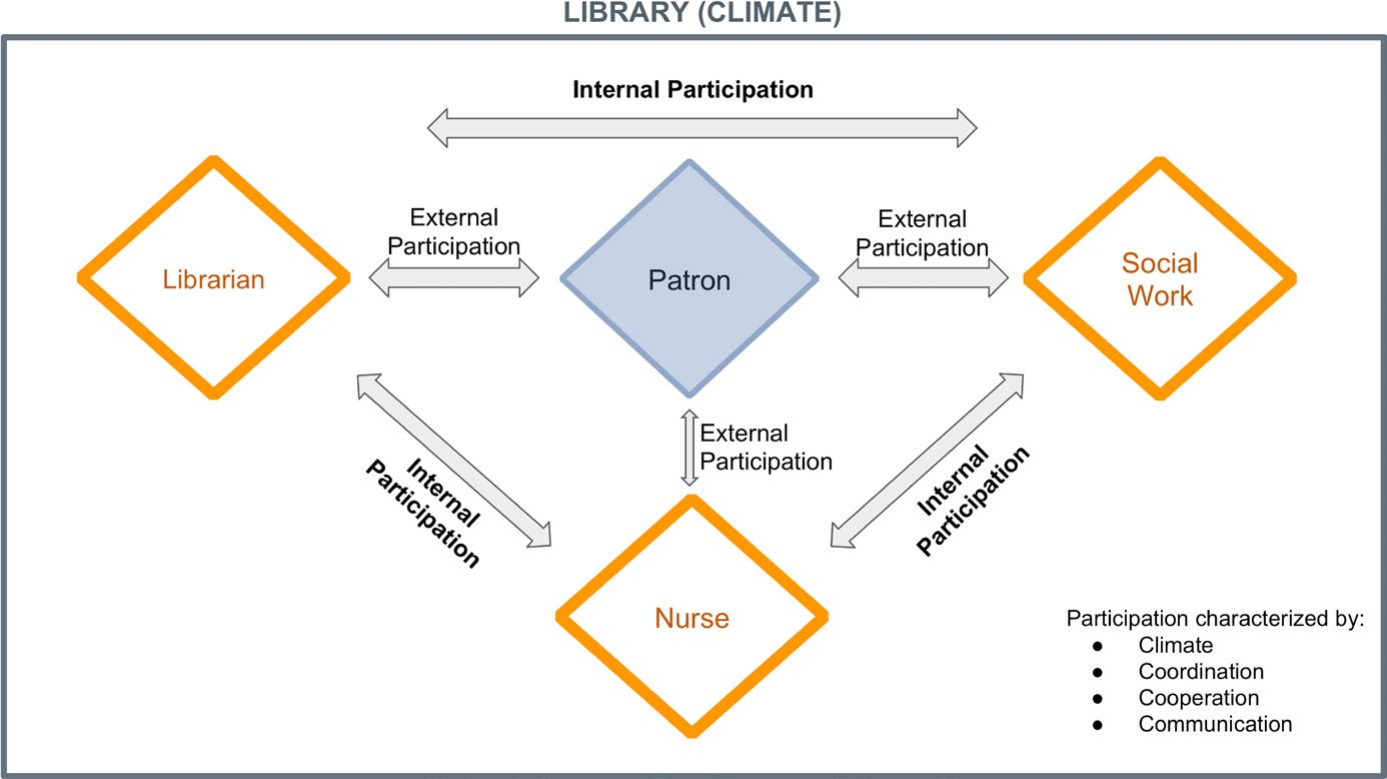
The model of patron‐centered interprofessional participation in the library [Color figure can be viewed at wileyonlinelibrary.com]

To address the health care access inequity for homeless persons in Philadelphia, the interprofessional model utilized three professions: nursing (registered nurse), social work (clinical social worker), and library science (a librarian or information specialist). The goal of the team was to connect the public with information and awareness of low or no‐cost health resources. The team of professionals operated under a patron‐centered interprofessional model (See Figure 1). The model informed the pilot project and was based upon patient‐centered interprofessional participation. This model focuses on interaction and participation between patron and professional (external participation) and participation within the interprofessional team (internal participation). The cornerstones of such participation are communication, cooperation, coordination, and working climate between patient and professional (Korner & Wirtz, 2013). With the library as the working climate, when the patron initiated interaction with one of the three professionals, they coordinated services and support, cooperated to meet patron needs, and communicated with each other and the patron. Each lent a different perspective to contribute to this patron‐centered interprofessional model.

Librarians are the experts in searching for information and sources to support literacy. They assist in building and supporting traditional reading English proficiency, job skills and literacy skills, such as numeracy, digital, and culinary literacy. Librarians play a crucial role in supporting health‐promoting behaviors and bolstering patron literacy. Low literacy is associated with numerous adverse health outcomes (Berkman, Sheridan, Donahue, Halpern, & Crotty, 2011). Because of their skill in addressing literateness and the link between literacy and health, librarians are well positioned to serve as health partners.

The social work role was a full‐time position, provided by a partnership with the City's Department of Behavioral Health. The social worker contributed knowledge and experience in managing complex social issues, such as housing status, food insecurity, social relationships, identification documents, mental health, and much more. As social workers, their unique focus on mental health literacy, that is, the belief and knowledge surrounding the recognition, management, and prevention of mental health disorders, were especially suited for the many library patrons dealing with mental health issues (Mendenhall & Frauenholtz, 2013). The presence of social workers in a community setting has a positive effect on those who are transitioning from different care settings, a common occurrence among the library patron population (Barber, Kogan, Riffenburgh, & Enguidanos, 2015). The social worker's ability to maintain a safe and open environment was essential to building rapport with patrons.

The registered nurse, provided by and grant supported by a large local academic nursing institution, practiced within the scope of community‐based nursing and couples his/her knowledge of disease prevention and health promotion with triage skills to address health and disease issues. Physical assessment skills allowed the nurse to address whether health concerns should be addressed in an ambulatory or emergency care setting. The health education expertise that the registered nurse provided enabled patrons to improve their health literacy and feelings of self‐efficacy, both shown to improve health outcomes (Adams, 2010; Lee, Lee, & Moon, 2016).

### 1.2 Intervention

Addressing the complex needs of the large homeless population at the library involved the interprofessional team delivering services. The social worker and registered nurse were on site at the library from 9 a.m. to 5 p.m., Monday to Friday, while a librarian was on site during all library hours of operation, 9 a.m.–9 p.m., Monday to Thursday, Friday and Saturday, 9 a.m.–5 p.m., and Sunday 1 p.m.–5 p.m. On these days, the social worker and the nurse would walk around the library to engage patrons who expressed social or health needs. When encountering patrons on these walking “rounds,” the nurse or social worker initiated a brief intake session to assess needs. Patron engagement often took the form of an informal conversation in the library's public spaces or at a desk in a private staff‐only area. While performing their daily duties at departmental desks, if a librarian encountered a patron whose needs were beyond their scope of expertise, they would consult with the registered nurse and the social worker to address those needs. Referrals to health care agencies or other community resources were made for patrons who expressed the need.

If necessary, the professional who first contacted the patron would consult with other team members to discuss needs and facilitate connection, that is, complete an internal “warm” transfer. The warm transfer consisted of either walking the patron over to one of the professionals in the building or calling via telephone. When warranted, a referral to external health or social service agency was made. These external community agency referrals included community health clinics, mental health providers, drug addiction rehabilitation facilities, free shower and laundry facilities, temporary shelters, meal sites, and housing agencies.

The referral process entailed confirming an available day and time, arranging transportation, calling and making appointments, sharing expectations of the appointment, and setting up a chance for a follow‐up in person or via telephone. Referral methods varied from case to case. For example, transportation could mean either obtaining a cab voucher or providing public transportation tokens, while calling and making an appointment could mean making a 15‐min appointment at a specific time or setting a timeframe within which a patron would be seen.

To track the completion of a referral, the registered nurse and social worker would follow up at least once a week either in person at the library, or through the phone. If patrons could not be contacted, the team would contact the referred agency directly in person or via phone to confirm the attendance of patrons with signed authorizations to disclose. This completion of a referral and confirmed access to the referred resource would be referenced as referral retention. Referral retention rate refers to the percentage of referrals that patrons completed and acted upon, for example, arrived at a scheduled appointment or met with a supportive housing agency, compared to total referrals made.

The information collection protocol was reviewed and approved by exemption by the Institutional Review Board of the academic nursing institution. Patron information was kept private and confidential. Community agencies and interprofessional team members did not share details of the actual appointment or meeting unless the patron opted to fill an authorization form to disclose health information.

## 2 RESULTS

The interprofessional care team jointly completed 470 visits, which included repeat visits from previously seen patrons. Table 1 shows some of the characteristics that were collected during these encounters. After accounting for repeat patrons, the team documented 358 unique patron engagements over a 9‐month period. The team documented the number of referrals and the type of referral, that is, external, internal, or both, with referrals categorized by housing status.

**Table 1:** Characteristics of the population served

Variable^a^	*N*	%^b^
Insurance status		
Uninsured	125	35
Insured	233	65
Housing status		
Stably house	260	72
Unstable housing in the past year	93	26
Incarceration involvement		
No interaction with a correction facility in the past year	338	94
Interaction with correction facility in the past year	20	6
Nativity status		
U.S. born	351	98
Immigrant	7	2

Note

This table describes the characteristics of the vulnerable population individuals who utilized the services of the interprofessional model (*N* = 358).

a Self‐identified information.

b Categories are not mutually exclusive, so percentages may not add up to 100.

Of those 358 engagements with nonrepeat patrons, 193 patrons (44%), received referrals to another in‐library professional and different health and social service agencies. The remaining 165 patrons (46%) either left before a referral was possible or declined the referral. Of the 193 patrons who were referred, 158 (82%) received referrals to external community health or social service organizations, 20 (10%) patrons were referred internally to other in‐library professionals, and 15 (8%) patrons received both an internal and subsequent external referral. Of those 158 external referrals, 89 patrons (56%) identified as homeless or unstably housed while 69 patrons (44%) identified as stably housed. Of the 20 patrons who were referred within the library, eight (40%) identified as stably housed, while 12 (60%) identified as unstably housed or homeless. Of the 15 patrons who received both an internal transfer and a proceeding referral to an external community resource, four (27%) patrons identified as stably housed, while 11 (73%) identified as unstably housed. Table 2 displays this breakdown of the type of referrals.

**Table 2:** Referral and referral retention rates

Intervention level	*N*	Completed referrals	Referral retention rate (%)
Total external referrals	158	90	57
Stably housed	69	13	19
Unstably housed	89	77	87
Total internal referrals	20	17	85
Stably housed	8	6	75
Unstably housed	12	11	92
Total internal and subsequent external	15	15	100
Stably housed	4	4	100
Unstably housed	11	11	100
Total	193	122	63

Note

This table exhibits the number of people that completed internal referrals, external referrals, or both (*N* = 193).

Of those patrons who received only external referrals, 57% (90 patrons) completed the external referral. When broken down into self‐identified categories of stably housed and unstably housed, the referral retention rate for those who were unstably housed was 87% (77 patrons) while the referral retention rate for those who were stably housed was 19% (13 patrons). Of those who received only internal referrals, the total referral retention rate was 85% (17 patrons). When split into housing status categories, 92% (11 patrons) of those unstably housed completed the internal referral while 75% (six patrons) of those who identified as stably housed completed the internal referral. For the patrons that received an internal and subsequent external referral, there was a 100% (15 patrons) referral retention rate for patrons who identified as stably housed and unstably housed or homeless.

Table 2 demonstrates the type of referrals made and the concordant referral retention rate for each housing category of patrons. The table shows that the referral retention rate is consistently greater than 87% for those who identified as unstably housed. The referral retention for those who are unstably housed rate is equal to or higher when compared to the referral retention rate for those who are stably housed. The highest rate (100%) was found among those who were referred both internally and then externally.

### 2.1 Needs for future study

These programmatic outcomes are limited because the patron's referral experience was not captured and evaluated. Similarly, the likelihood with which they would return or send others to the referred agency was not recorded. Other predictors of the quality of these referrals, such as self‐reported health status, number of previous primary care visits, sociodemographic information, and experience with previous health care providers, were not assessed.

It is also difficult to control for other variables that could have exacerbated or alleviated the difficulty with which the patron completed the referral. These confounding variables include assessments of the social support structure and previous engagement with the health care system. Further, the team was unable to collect pre‐intervention data, as most participants were unwilling to participate due to lack of relationship development. Future studies should consider the inclusion and analysis of pre‐intervention data and measures of patron and professional experience with the patron‐centered interprofessional model.

### 2.2 Discussion and implications

The homeless population is at risk for missing appointments, not receiving adequate outpatient care, utilizing the emergency room at high rates and having frequently no‐show rates (Chang, Sewell, & Day, 2015; Hwang, Weaver, Aubry, & Hoch, 2011; Hwang, Wilkins, et al., 2011; Williamson, Ellis, Wilson, Mcqueenie, & Mcconnachie, 2017). These missed appointments and no‐shows cost a significant amount to clinics and other health care facilities (Kheirkhah, Feng, Travis, Tavakoli‐Tabasi, & Sharafkhaneh, 2015). The consistently higher referral retention rate with the internal and subsequent external referral, when compared to referral retention rate of those stably housed, this pilot project provides some evidence to support that having multiple professions co‐located in one accessible place may decrease the number of no‐show appointments and increase the use of community and health resources.

This model has financial implications for public health, with the potential to reduce no‐show costs and reduce the burden of high emergency room utilization. Financial analysis of these effects has not been included in this study. The high referral retention rate suggests that an interprofessional care team may play a part to facilitate referral follow‐ups, encourage primary care use over emergency room use, and to promote earlier ambulatory visits to manage chronic physical and mental health diseases.

The reason for the high referral retention rate was not investigated. However, potential explanations may be found in the strengths of the interprofessional model. Each profession's unique background allows for (1) a more comprehensive range of referral resources, (2) greater accountability, and (3) a broader network with which the patron could build rapport. Further research and more focused data analysis are necessary to understand the relationship of increased referral retention rate and the implementation of the interprofessional model (Franklin, Bernhardt, Lopez, Long‐Middleton, & Davis, 2015).

Although the rationale behind the high referral retention rate was not explicitly researched, the co‐location of services in the library setting was pivotal to the success of the model. The free and public nature of the library situates itself as a critical community access point. Furthermore, its reputation as a neutral resource‐filled space and its centrally convenient location makes it an anchor point, both physically and socially. This present paper creates further opportunity to bolster the efficacy of place‐based health care delivery (Dankawa‐Mullan & Perez‐Stable, 2016).

The success of its outcomes is supported by reports of positive health outcomes with interprofessional teamwork, especially in the management of chronic diseases (Franklin et al., 2015; Morgan, Pullon, & McKinlay, 2015). The open communication between the three professions allowed for sharing of comprehensive information. This information sharing allowed the professionals to navigate the patron's complex needs and subsequently complete the referrals, which might otherwise be missed by a single professional. The 100% referral retention rate for internal and subsequent external referrals speaks to the potential impact of the increased support provided by the interprofessional model. This pilot project lays the groundwork for further work surrounding the possible effect interprofessional teams may have on patient outcomes and behavior.

## 3 CONCLUSION

The pilot intervention and preliminary results of an increased retention referral rate demonstrate the opportunity to integrate creativity and collaboration when implementing methods to help patrons gain entry into and remain in the continuum of care. In a time where determinants of access to care are so numerous and variant, this pilot suggests that the use of multiple professionals across disciplines allows for the sharing of unique perspectives that can address these manifold barriers. Future interventions should consider innovative interprofessional models in addressing traditional health care access problems for vulnerable populations such as the homeless.

## CONFLICT OF INTEREST

The author has no conflicts of interest or relationships to disclose.

## References

[phn12561-bib-0001] Adams, R. J. (2010). Improving health outcomes with better patient understanding and education. Risk Management and Healthcare Policy, 3, 61–72. 10.2147/RMHP.S7500 22312219 PMC3270921

[phn12561-bib-0002] Agency for Healthcare Research and Quality (2015). *2015 National Healthcare Quality and Disparities Report and 5th Anniversary Update on the National Quality Strategy* . Retrieved from https://www.ahrq.gov/research/findings/nhqrdr/nhqdr15/access.html

[phn12561-bib-0003] Altarum Institute (2017). *Altarum Institute Center for Sustainable Health Spending Health Sector Trend Report, May 2017* . Retrieved from https://altarum.org/sites/default/files/uploaded-publication-files/Altarum%20RWJF%20Trend%20Report%20May%202017.pdf

[phn12561-bib-0004] Barber, R. D. , Kogan, A. C. , Riffenburgh, A. , & Enguidanos, S. (2015). A role for social workers in improving care setting transitions: A case study. Social Work Health Care, 54(3), 177–192. 10.1080/00981389.2015.1005273 PMC5479582 25760487

[phn12561-bib-0005] Berkman, N. D. , Sheridan, S. L. , Donahue, K. E. , Halpern, D. J. , & Crotty, K. (2011). Low health literacy and health outcomes: An updated systematic review. Annals of Internal Medicine, 155(2), 97–107. 10.7326/0003-4819-155-2-201107190-00005 21768583

[phn12561-bib-0006] Chang, J. T. , Sewell, J. L. , & Day, L. W. (2015). Prevalence and predictors of patient no‐shows to outpatient endoscopic procedures scheduled with anesthesia. Gastroenterology, 148(4), S590 10.1016/s0016-5085(15)31999-5 PMC4589132 26423366

[phn12561-bib-0007] Corrigan, P. , Pickett, S. , Kraus, D. , Burks, R. , & Schmidt, A. (2015). Community‐based participatory research examining the health care needs of African Americans who are homeless with mental illness. Journal of Health Care for the Poor and Underserved, 26, 119–133. 10.1353/hpu.2015.0018 25702732 PMC5388444

[phn12561-bib-0008] Dankwa‐Mullan, I. , & Pérez‐Stable, E. J. (2016). Addressing health disparities is a place‐based issue. American Journal of Public Health, 106(4), 637–639. 10.2105/AJPH.2016.303077 26959267 PMC4816016

[phn12561-bib-0009] Donley, A. M. , & Wright, J. D. (2017). The health of the homeless. Sociology Compass, 12(1), e12550 10.1111/soc4.12550

[phn12561-bib-0010] Fazel, S. , Geddes, J. R. , & Kushel, M. (2014). The health of homeless people in high‐income countries: Descriptive epidemiology, health consequences, and clinical and policy recommendations. Lancet, 384, 1529–1540. 10.1016/s0140-6736(14)61132-6 25390578 PMC4520328

[phn12561-bib-0011] Franklin, C. M. , Bernhardt, J. M. , Lopez, R. P. , Long‐Middleton, E. R. , & Davis, S. (2015). Interprofessional teamwork and collaboration between community health workers and healthcare teams: An integrative review. Health Services Research and Managerial Epidemiology, 2, 1–7. 10.1177/2333392815573312 PMC5266454 28462254

[phn12561-bib-0012] Free Library of Philadelphia (2016). *Free Library of Philadelphia 2016 Annual Report* . Retrieved from https://libwww.freelibrary.org/assets/pdf/about/annualreport/annualreport2016.pdf

[phn12561-bib-0013] Horrigan, J. B. (2015). Libraries at the crossroads. Washington, DC: Pew Research Center Retrieved from https://www.pewinternet.org/2015/09/15/libraries-at-the- crossroads /

[phn12561-bib-0014] Hwang, S. W. , Weaver, J. , Aubry, T. , & Hoch, J. S. (2011). Hospital costs and length of stay among homeless patients admitted to medical, surgical, and psychiatric services. Medical Care, 49(4), 350–354. 10.1097/MLR.0b013e318206c50d 21368678

[phn12561-bib-0015] Hwang, S. W. , Wilkins, E. , Chambers, C. , Estrabillo, E. , Berends, J. , & Macdonald, A. (2011). Chronic pain among homeless persons: Characteristics, treatment, and barriers to management. BMC Family Practice, 12(1), 73 10.1186/1471-2296-12-73 21740567 PMC3141516

[phn12561-bib-0016] Jabrera Mesa, M. L. , Morales Asencio, J. M. , Rivas Ruiz, F. , & Porras González, M. H. (2017). Análisis del coste económico del absentismo de pacientes en consultas externas [Analysis of economic cost of missed outpatient appointments]. Revista De Calidad Asistencial, 32(4). 10.1016/j.cali.2017.01.004. 28476506

[phn12561-bib-0017] Johnson, P. (2015). Homeless, not hopeless: A view from the Free Library. *WHYY*: Retrieved from. https://whyy.org/articles/homeless-not-hopeless-a-view-from-the-free-library/

[phn12561-bib-0018] Kheirkhah, P. , Feng, Q. , Travis, L. M. , Tavakoli‐Tabasi, S. , & Sharafkhaneh, A. (2015). Prevalence, predictors and economic consequences of no‐shows. BMC Health Services Research, 16(13). Retrieved from: https://bmchealthservres.biomedcentral.com/articles/10.1186/s12913-015-1243-z 10.1186/s12913-015-1243-z PMC4714455 26769153

[phn12561-bib-0019] Korner, M. , & Wirtz, M. A. (2013). Development and psychometric properties of a scale for measuring internal participation from a patient and health care professional perspective. BMC Health Services Research, 13, 374 10.1186/1472-6963-13-374 24083632 PMC3850532

[phn12561-bib-0020] Lee, E.‐H. , Lee, Y. W. , & Moon, S. H. (2016). A structural equation model linking health literacy to self‐efficacy, self‐care activities, and health‐related quality of life in patients with type 2 diabetes. Asian Nursing Research, 10(1), 82–87. 10.1016/j.anr.2016.01.005 27021840

[phn12561-bib-0022] Mendenhall, A. N. , & Frauenholtz, S. (2013). Mental health literacy: Social work’s role in improving public mental health. Social Work, 58(4), 365–368. 10.1093/sw/swt038 24450023

[phn12561-bib-0023] Mitchell, M. S. , León, C. L. K. , Byrne, T. H. , Lin, W.‐C. , & Bharel, M. (2017). Cost of health care utilization among homeless frequent emergency department users. Psychological Services, 14(2), 193–202. 10.1037/ser0000113 28481604

[phn12561-bib-0024] Morgan, S. , Pullon, S. , & McKinlay, E. (2015). Observation of interprofessional collaborative practice in primary care teams: An integrative literature review. International Journal of Nursing Studies, 52(7), 1217–1230. 10.1016/j.ijnurstu.2015.03.008 25862411

[phn12561-bib-0025] Office of Homeless Services (2017). *Point‐in‐Time Count PA‐500 Philadelphia CoC* . Retrieved from https://www.philadelphiaofficeofhomelessservices.org/wp-content/uploads/2017/09/2017-hdx-point-in-time-count-summary.pdf

[phn12561-bib-0026] Philadelphia Department of Public Health (2014). *Community Health Assessment* . Retrieved from https://www.phila.gov/health/pdfs/CHAreport_52114_final.pdf

[phn12561-bib-0027] U.S. Department of Housing and Urban Development . (2017). *The 2017 annual homeless assessment report to congress* . Retrieved from https://www.hudexchange.info/resources/documents/2017-AHAR-Part-1.pdf

[phn12561-bib-0028] Williamson, A. E. , Ellis, D. A. , Wilson, P. , Mcqueenie, R. , & Mcconnachie, A. (2017). Understanding repeated non‐attendance in health services: A pilot analysis of administrative data and full study protocol for a national retrospective cohort. British Medical Journal Open, 7(2), e014120 10.1136/bmjopen-2016-014120 PMC5319001 28196951

